# Longer TOMM40 poly-T variants associated with higher FDDNP-PET medial temporal tau and amyloid binding

**DOI:** 10.1371/journal.pone.0208358

**Published:** 2018-12-05

**Authors:** Prabha Siddarth, Alison C. Burggren, David A. Merrill, Linda M. Ercoli, Zanjbeel Mahmood, Jorge R. Barrio, Gary W. Small

**Affiliations:** 1 Department of Psychiatry & Biobehavioral Sciences, Semel Institute for Neuroscience & Human Behavior, David Geffen School of Medicine at UCLA, Los Angeles, United States of America; 2 Center for Cognitive Neurosciences, UCLA, Los Angeles, United States of America; 3 Lewis Center for Neuroimaging, The University of Oregon, Eugene, United States of America; 4 San Diego State University/University of California, San Diego Joint Doctoral Program in Clinical Psychology, United States of America; 5 Department of Molecular & Medical Pharmacology, UCLA, Los Angeles, United States of America; Nathan S Kline Institute, UNITED STATES

## Abstract

**Background:**

The translocase of outer mitochondrial membrane 40 (TOMM40), which lies in linkage disequilibrium with the apolipoprotein E (APOE) gene, has been implicated in Alzheimer’s disease (AD). TOMM40 influences AD pathology through mitochondrial neurotoxicity, and the medial temporal lobe (MTL) is the most likely brain region for identifying early manifestations of AD-related morphology changes. While early reports indicated that the longer length poly-T allele of TOMM40 increases risk for AD, these findings have not been consistently replicated in further studies. We examined the effect of TOMM40 and APOE on regional brain positron emission tomography (PET) 2-(1-{6-[(2 [F18]fluoroethyl) (methyl) amino]-2-naphthyl}ethylidene)malononitrile (FDDNP) binding values in MTL.

**Methods:**

A total of 73 non-demented older adults (42 females; mean age: 62.9(10.9) completed genotyping for both APOE and TOMM40 and received FDDNP-PET scans. For TOMM40, the lengths of the poly-T sequence were classified as short (14–20 repeats; S), long (21–29 repeats, L) or very long (>29 repeats, VL). Using general linear models, we examined medial temporal lobe FDDNP binding and cognitive functioning between TOMM40 and APOE-4 groups, with age, sex, and education as covariates.

**Results:**

Data from 30 individuals with APOE-4 and L TOMM40 poly-T length, 11 non E4 TOMM40 S/S, 14 non E4 TOMM40 S/VL and 13 non E4 TOMM40 VL/VL were analyzed. Medial temporal FDDNP binding differed significantly between TOMM40/APOE groups (F(3,62) = 3.3,p = .03). Participants with TOMM40 S/S exhibited significantly lower binding compared to TOMM40 S/VL and APOE-4 carriers. We did not find a significant relationship between TOMM40 poly-T lengths/APOE risk groups and cognitive functioning.

**Conclusions:**

This is the first report to demonstrate a significant association between longer TOMM40 poly-T lengths and higher medial temporal plaque and tangle burden in non-demented older adults. Identifying biomarkers that are risk factors for AD will enhance our ability to identify subjects likely to benefit from novel AD treatments.

## Introduction

Alzheimer's disease (AD) is the most common form of dementia and is a heterogeneous disorder with environmental and genetic components. Several non-genetic risk factors for AD (besides age), including hypertension, estrogen supplements, smoking, stroke, heart disease, depression, arthritis, and diabetes as well as protective factors such as exercise, healthy diet, intellectual and social engagement have been identified [1–5]. Several genetic mutations have been implicated in familial (primarily early-onset) AD; however, for the more common late onset AD, while genome wide association studies have detected a number of single nucleotide polymorphisms [6–9], most of these polymorphisms, apart from the apolipoprotein E (APOE) gene, have a small effect on AD risk [10]. While the APOE gene has been consistently pinpointed as the primary risk gene, possession of the E4 variant of the APOE gene is by itself not sufficiently powerful to identify those likely to develop AD with high accuracy [9, 11]. More recently, polygenic approaches [12, 13] have been developed that yield genetic risk scores incorporating AD associated single nucleotide polymorphisms, and these show some promise in identifying genetic risk for AD beyond APOE, even though the results were mixed in predicting AD conversion in participants with Mild Cognitive Impairment.

Using phylogenetic analysis, Roses and co-workers have implicated the translocase of outer mitochondrial membrane 40 (TOMM40), which lies in linkage disequilibrium with APOE, in the development of AD [14–16]. This stretch of DNA varies with respect to the length of a poly-T polymorphism. Longer length poly-T variants were found to be associated with increased risk for AD, as well as a lower age at onset of dementia. However, further research on TOMM40’s risk for AD was inconclusive, with some studies showing an association with AD in the absence of APOE-4 [17]; and some reports indicating no correlation between TOMM40 poly-T repeat length and age at dementia onset [18,19]; and still others showing that increasing length was associated with a lower risk of AD [20]. It has also been pointed out that since TOMM40 is in such close linkage disequilibrium with APOE, any signal at the TOMM40 locus may be confounded with the APOE signal [21, 22]. Investigators have therefore examined the effect of TOMM40 poly-T repeat length stratified by APOE genotype or specifically within APOE-4 non-carriers [23, 24]. Notwithstanding these few studies, the effects of TOMM40 and APOE4 on AD pathology remain to be elucidated.

The main neuropathological hallmarks of AD are senile plaques and neurofibrillary tangles. In recent years, positron emission tomography (PET) ligands have been developed for measuring *in vivo* AD pathology in the brain and have been shown to be useful in clinical use and for patient recruitment and as an outcome measure in clinical trials [25–27]. Notably, our group developed a small molecule, 2-(1-{6-[(2-[F-18]fluoroethyl)(methyl)amino]-2-naphthyl}ethylidene) malononitrile (FDDNP), for use as an *in vivo* chemical marker of cerebral aggregates of Aβ and tau proteins [28]. FDDNP-PET provides a measure of both plaque and tangle binding levels in the living human brain, and the *in vivo* distribution of FDDNP in the brain follows patterns of plaque and tangle distribution observed at autopsy [29, 30]. Also, FDDNP binding levels have previously been shown to correlate with cognitive function in older adults [29] and be predictive of cognitive decline [31] in non-demented individuals, making it a valuable biomarker to study early AD-related changes in the brain.

In this study, we examined a cohort of non-demented older adults to determine the effect of TOMM40 and APOE4 on FDDNP binding values in the medial temporal lobe (MTL), a region showing high concentrations of tau and Aβ even before patients develop dementia. We chose to study non-demented individuals in order to identify possible genetic biomarkers using sensitive imaging techniques that can pinpoint early manifestations of pathophysiological changes in the brain. We focused on the MTL since the atrophy and anti-neuroplastic processes occurring in AD-related cognitive decline are recognized to begin in the MTL, and global MTL volume atrophy is known to be associated with memory impairment and AD [32].

## Methods

### Participants

A total of 73 non-demented older adults who had genotype testing and FDDNP-PET scans were drawn from a larger study of predictors of cognitive decline. Data were collected between December 2001 and January 2009. Briefly, volunteers from the community were recruited through advertisements, media coverage of the study, and referrals by physicians and families. Members of the research staff screened potential volunteers via telephone interviews. All subjects underwent FDDNP-PET scans, as well as clinical and cognitive assessments performed by investigators who were blinded to the results of FDDNP-PET scans. The study was reviewed and approved by the UCLA Human Subjects Protection Committee and participants gave written informed consent according to the UCLA Human Subjects Protection Committee procedures. Cumulative radiation dosimetry for all scans was below the mandated maximum annual dose and in compliance with state and federal regulations. Exclusion criteria included MRI intolerance, evidence of stroke or brain tumor on MRI, traumatic brain injury, cognitively-altering medications, and excessive head motion during scanning. Participants with a diagnosis of Alzheimer’s disease or other dementias were also excluded. Subjects were also excluded for any history of alcohol or substance abuse, head trauma or other major systemic disease affecting brain function, a history of neurological or psychiatric disorders, as well as hypertension or cardiovascular disease.

During study intake, participants underwent an extensive physical and medical examination, laboratory screening including blood tests to rule out medical conditions that could affect cognitive performance, and a medical history assessment. The current study was conducted on a subset of 73 of these participants who had successfully completed genotyping for both APOE and TOMM40, as well as imaging procedures.

### DNA sampling and genotyping

DNA was extracted from blood. Samples were aliquoted on 96-well plates for determination of both APOE and TOMM40 genotypes. Genotyping for the APOE gene was done by the UCLA Center for Neurobehavioral Genetics using standard methods [33]. Genotyping for TOMM40 using the rs10524523 (‘523’) allele was completed at Polymorphic DNA Technologies (Alameda, CA, USA; http://www.polymorphicdna.com). TOMM40 polymorphisms were analyzed using polymerase chain reaction (PCR) and bidirectional direct Sanger sequencing of the DNA templates on an Applied Biosystems 3730xl DNA Analyzer (Applied Biosystems Inc., Carlsbad, CA) followed by sequence data analysis. This polymorphism, 523, is a homopolymer length polymorphism (poly-T) located in an intronic region of TOMM40. The poly-T lengths for each chromosome were converted into the S, L, and VL standard labeling [15].

### Imaging methods

FDDNP was prepared at very high specific activities (>37 GBq/mol), as described in detail elsewhere [34]. All scans were performed with the ECAT HR or EXACT HR+ tomograph (Siemens-CTI, Knoxville, TN) with subjects supine and the imaging plane parallel to the orbito meatal line. A bolus of FDDNP (320–550 MBq) was injected via an indwelling venous catheter, and consecutive dynamic PET scans were performed for 2 hours. Scans were decay corrected and reconstructed using filtered back-projection (Hann filter, 5.5mm FWHM) with scatter and measured attenuation correction. The resulting images contained 47 contiguous slices with plane separation of 3.37mm (ECAT HR) or 63 contiguous slices with plane separation of 2.42mm (EXACT HR+). Determinations of data reproducibility were performed when the new scanner was introduced in the Nuclear Medicine clinic using phantoms and comparing results between scanners. Nonparametric Wilcoxon two-sample tests found no significant differences in regional FDDNP signals between the two PET scanners.

All subjects received MRI scans that were co-registered to PET scans for determination of ROIs. These anatomical brain scans were obtained using either a 1.5 T or 3 T magnet (General Electric-Signa, Milwaukee, WI) scanner. Fifty-four transverse planes were collected throughout the brain, superior to the cerebellum, using a double-echo, fast-spin echo series with a 24-cm field of view and 256 x 256 matrix with 3 mm/0 gap (TR = 6000 [3 T] and 2000 [1.5 T]; TE = 17/85 [3 T] and 30/90 [1.5 T]). Rules for ROI drawing were based on the identification of gyral and sulcal landmarks with respect to the atlas of Talairach and Tournoux [35]. All PET and MRI scans were read and the ROIs were drawn by investigators blind to clinical assessments. Previous inter-rater reliability studies have confirmed high consistency and reliability using this method [36].

FDDNP-PET binding levels were quantified as previously described [29]. Briefly, we performed Logan graphical analysis with cerebellum as the reference region for time points between 30 and 125 minutes [37]. The slope of the linear portion of the Logan plot is the relative distribution volume (DVR), which is equal to the distribution volume of the tracer in an ROI divided by that in the reference region. We generated DVR parametric images and analyzed them using gray matter ROIs drawn manually on the FDDNP-PET image obtained in the first 5 minutes after injection (the perfusion image). This image shows the perfusion pattern and has sufficient anatomical information to identify the cerebellum and cerebellar gray matter. ROIs were drawn bilaterally on the medial temporal (containing limbic regions, including hippocampus, parahippocampal, and entorhinal areas) region, as previously described [38] and was expressed as an average of left and right regions. MTL binding from an FDDNP-PET scan was the single pathology score used for each participant in this study.

### Neuropsychological testing

A neuropsychological test battery was administered to assess specific cognitive domains: 1) Memory, including the Wechsler Memory Scale Third Edition (WMS-III) logical memory (delayed score), and Buschke selective reminding (delayed score); 2) Language, including the Boston naming test and letter (F.A.S.) and category (Animal naming test) fluency; 3) Attention and information-processing speed, including Trail making task A, Stroop color naming (Kaplan version), and Wechsler Adult Intelligence Scale Third Edition (WAIS-III) digit symbol; and 4) Executive functioning, including Trail making task B, and Stroop Interference (Kaplan version). We converted raw test scores to Z scores by standardizing them to a mean of 0 and a standard deviation of 1. We computed domain Z scores by averaging those Z scores belonging to the cognitive tests in that domain.

### Statistical analyses

Data were screened for outliers for all variables included in these analyses. For TOMM40, the lengths of the poly-T sequence were classified as short (14–20 repeats; i.e. ‘S’), long (21–29 repeats, i.e., ‘L’) or very long (>29 repeats, i.e., ‘VL’), as has been done in the literature [15]. For APOE, we categorized subjects into two groups: those carrying at least one E4 allele and those without any E4 allele. Since the TOMM40 L variant is almost exclusively linked to the APOE-4 allele and the VL and S variants are in strong linkage disequilibrium with APOE-3, we further classified participants into the following 4 groups: TOMM40 S/S, TOMM40 S/VL and TOMM40 VL/VL (excluding those with APOE-4 allele), and APOE-4 carriers.

Demographic and clinical measures were compared between groups using Kruskal-Wallis tests for continuous measures and Fishers exact tests for categorical measures. Nonparametric ANCOVAs (using ranked MTL FDDNP binding levels rather than raw DVR values) with Tukey-Kramer adjusted post-hoc comparisons were used to test for statistically significant differences in MTL FDDNP binding among the four subject groups. For cognitive performance, we estimated a similar nonparametric MANCOVA with cognitive domain scores as dependent variables. For both these models, age, sex, educational level and Mini Mental State Examination (MMSE; not used for cognitive models) scores were evaluated and retained as covariates, if found necessary. In addition to the standard statistics, effect size (ES; Cliff’s delta [39]) estimates are also presented. A significance level of p < 0.05 (two-tailed) was used for all inferences.

## Results

**Fig 1:**
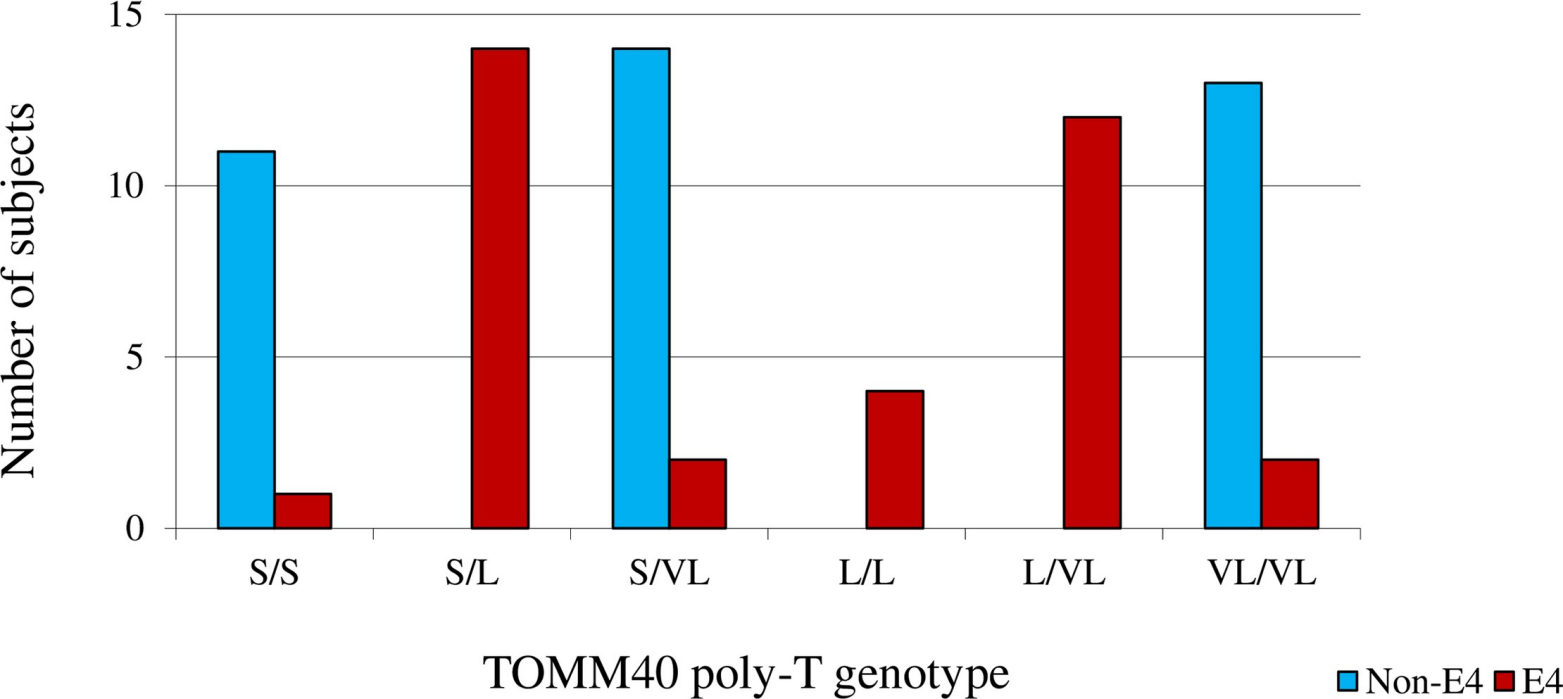
Distribution of TOMM40 variants by APOE status.

Among the 73 participants (42 (57.5%) females; mean ± SD age: 62.9 ± 10.9; MMSE: 28.9 ± 1.2), thirty-five individuals posessed a copy of the E4 allele. Of these 35 subjects, 5 participants had TOMM40 poly-T lengths not classified as L (they were 1 S/S, 2 S/VL, and 2 VL/VL) and thus were not used in further analyses. Among the 38 non APOE-4 carriers, 11 were classified as S/S, 14 as S/VL and 13 as VL/VL (Fig 1). There were no differences in demographic variables across the TOMM40 and APOE-4 groups, including age, sex, educational level, ethnicity and MMSE (Table 1).

**Table 1:** Demographic characteristics of subject groups.

Measure^	Non E4 S/S	Non E4 S/VL	Non E4 VL/VL	E4*	Statistics, p-value^#^
	(N = 11)	(N = 14)	(N = 13)	(N = 30)	
Females	6 (54.6)	10 (71.4)	8 (61.5)	14 (46.7)	0.5
Age, years	62 (46–85)	62 (46–82)	62 (46–85)	67 (46–87)	6.3, 0.1
Education, years	18 (13–22)	17 (14–22)	16.(11–20)	18 (14–22)	1.8, 0.6
Ethnicity					0.3
Caucasian	9 (81.8)	13 (92.9)	12 (92.3)	27 (90.0)	
African-American	0 (0)	0 (0)	0 (0)	1 (3.3)	
Asian	2 (18.2)	0 (0)	0 (0)	2 (6.7)	
Other	0 (0)	1 (7.1)	1 (7.7)	0 (0)	
MMSE^$^	29 (27–30)	30 (28–30)	29 (27–30)	29 (26–30)	5.3, 0.2

^Values are medians with range in parentheses, or number of subjects with percentage (%) in parentheses

*5 E4 participants with S/S (1), S/VL (2) and VL/VL (2) TOMM40 poly-T lengths not included

^#^Kruskal-Wallis test statistics and p-values for continuous measures; Fisher’s exact p-value for categorical measures

^$^Mini Mental State Examination

**Fig 2:**
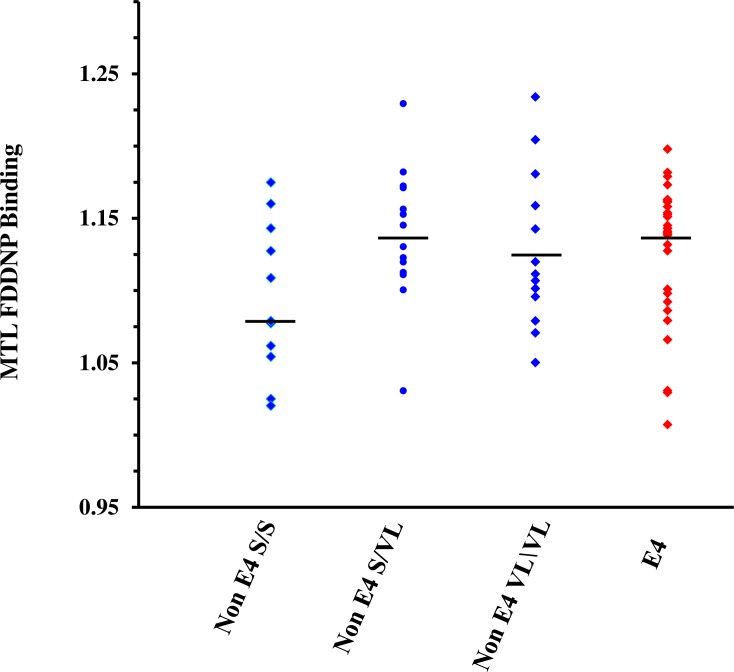
Medial temporal FDDNP-PET binding by non APOE-4 TOMM40 variants and APOE-4 carriers. Non APOE-4 participants with the TOMM40 S/S variant (median M = 1.08, interquartile range IQR = .09) exhibited significantly lower binding compared to non APOE-4 S/VL (M = 1.14, IQR = .06, t(62) = 3.1, p = .004; effect size ES = .47) and compared to APOE-4 carriers (M = 1.14, IQR = .06, t(62) = 2.4, p = .02; ES = .39).

Analyses revealed a significant association between medial temporal FDDNP binding and TOMM40/APOE-4 groups (F(3,62) = 3.3, p = .03) (Fig 2). Non APOE-4 participants with the TOMM40 S/S variant (median M = 1.08, interquartile range IQR = .09, range 1.02–1.17) exhibited significantly lower binding compared to non APOE-4 S/VL (M = 1.14, IQR = .06, range 1.03–1.23, t(62) = 3.10, p = .004; ES = .47) and compared to APOE-4 carriers (M = 1.14, IQR = .06, range 1.01–1.20, t(62) = 2.4, p = .02; ES = .39). No other pair-wise differences reached statistical significance. We also did not find a significant relationship between TOMM40 poly-T lengths/APOE risk groups and cognitive scores in any of the domains of cognitive function (multivariate F(12,140) = 0.7, p = .7; univariate p-values range from .5 to .9).

## Discussion

To our knowledge, this is the first report to identify significant associations between TOMM40 poly-T lengths and higher medial temporal plaque and tangle burden in the living brain of non-demented older adults within individuals not carrying the APOE-4 allele. Participants with the shortest TOMM40 poly-T lengths had significantly less plaque and tangle burden in the medial temporal lobe compared to both those with longer TOMM40 lengths and those with the APOE-4 genetic risk. In contrast, those subjects with longer TOMM40 poly-T repeat lengths, who do not carry the E4 allele, had comparable MTL plaque and tangle burden to the APOE-4 carriers. We did not, however, detect a relationship of TOMM40/APOE risk factors with cognitive performance in this cohort. It is possible that in this relatively small but highly educated cohort, we lacked statistical power to detect an association of cognitive functioning with the genetic risk factors, and that the FDDNP imaging measures are a more sensitive indicator of the changes occurring in the brain.

The apolipoprotein E4 variant on chromosome 19 has historically been the most significant genetic marker for AD. Other genetic risk factors have been identified using genome-wide association studies, but have mostly not been replicated in subsequent studies or had relatively small effects. To date, TOMM40 is the only gene identified that is thought to contribute to late onset AD-related mitochondria dysfunction [40]; however, it has been suggested that the statistically significant correlation of TOMM40 with AD risk is due to linkage disequilibrium with APOE on chromosome 19. In agreement with previous reports [41, 42], the majority of the E4 cohort in our sample (85.7%) possessed at least one ‘long’ TOMM40 variant, and the non-E4 cohort was either ‘short’ (65.8%) or ‘very long’ (34.2%).

Our finding that longer TOMM40 poly-T lengths, in the absence of APOE genetic risk, is associated with a greater degree of plaques and tangles in the brain is consistent with our previous study [23] that demonstrated cortical thinning in MTL sub-regions in subjects with no APOE risk, but elevated TOMM40 risk. Further, a recent study [43] that examined the association between verbal memory and 1.2 million gene variations across the human genome, showed that only TOMM40 had a strong link to declines in both immediate recall and level of delayed recall, and further, found an independent effect of TOMM40 among individuals who do not carry the APOE E4 allele. Several previous studies have also found that TOMM40 is associated with hippocampal atrophy [44] and decline in cognitive performance [45], independently of APOE. Indeed, even the studies that did not yield an APOE-independent effect of TOMM40 on AD risk found an association between TOMM40 and AD risk within the E3/E3 participants [17, 20]. It has also been suggested [40] that the effects of the longer variants of the TOMM40 genotype may be specific to non-symptomatic individuals, or present only in very early stages of the disease. Our results are consistent with this hypothesis and further emphasize the need for examining the effect of TOMM40 risk in individuals before the onset of dementia symptoms.

Methodologic limitations should be noted. First, this is a cross-sectional, observational study, which cannot infer causality. The sample size is limited and the number of individuals with the S/S variant was in particular small. These findings will thus need to be replicated in larger data sets. The participants carrying the E4 allele were older than the other groups, though the difference was not statistically significant. Further, we controlled for age in all our analyses, but it is still possible that some of the observed effects were due to this difference in age. FDDNP binding could also be affected by other variables, such as cerebrovascular risk, that were not taken into account in the present analyses. We also did not observe a relationship between cognitive performance and genetic risk groups, which may be due the small sample size. Advantages of this study are application of an AD neuropathology specific imaging tracer to a well-characterized cohort to examine AD genetic risk. It should be noted that neuropathology in the MTL in AD is predominantly composed by tau aggregates and relatively less predominant Aβ [46] and FDDNP MTL binding levels reflect this distribution, as also shown by neuropathology autopsy determinations [29].

AD is a highly heterogeneous disorder, and neither genetic nor imaging markers alone are likely to be useful in definitively predicting who will develop AD. The current finding that FDDNP binding is related to a genetic risk factor for AD may imply that there are changes in the brain that may be phenotypic in prodromal AD. It is intriguing to consider the possibility of developing an AD risk score for individuals based on genetic, neuroimaging and lifestyle factors. Further research is required to integrate and verify the existing results before such a score can be validated and applied clinically. However, identifying biomarkers that are risk factors for AD will enhance our ability to identify subjects likely to benefit from the novel AD treatments currently under development.

## Supporting information

S1 Table Demographic, genetic and FDDNP binding measures of all participants. (XLSX)
